# Indirect effects of long-term care insurance: does it affect the hospital expenditures of ineligible disabled individuals

**DOI:** 10.3389/fpubh.2025.1687682

**Published:** 2025-11-05

**Authors:** Yanling Yi, Jing Xin, Junxia Liu

**Affiliations:** ^1^School of Business Administration, Guizhou University of Commerce, Guiyang, China; ^2^School of Public Administration, Zhongnan University of Economics and Law, Wuhan, China

**Keywords:** long-term care insurance, medical expenditures, indirect effects, ineligible disabled individuals, China

## Abstract

**Introduction:**

The literature on the impacts of long-term care insurance (LTCI) on medical expenses has primarily focused on beneficiaries or all older adults, leaving theoretical analysis and the effects of LTCI on the ineligible group unexplored. This study investigates the indirect effects of LTCI on the hospital expenditures of disabled individuals who are ineligible for benefits in China.

**Methods:**

Based on Becker’s household production function, we construct a theoretical model to analyze the impacts of LTCI on the hospital expenditures of disabled individuals, both eligible and ineligible. Furthermore, we leverage a quasi-experimental design focusing on the regional variation in the rollout of LTCI in the first round of national pilot cities and employ a difference-in-difference (DID) approach to identify the causal effects of LTCI on the hospital expenditures of ineligible disabled individuals, using the unbalanced panel data combined with four waves’ survey data of China Health and Retirement Longitudinal Study (CHARLS) in 2011, 2013, 2015, and 2018 and the corresponding years’ statistical data of China Urban Statistical Yearbook.

**Results:**

Theoretically, we find that LTCI will affect the hospital expenditures of ineligible disabled individuals through a negative substitution effect, a positive output effect, and a negative health effect, just as the effects observed in their eligible counterparts, leading to an ambiguous total effect. Empirically, we demonstrate that the implementation of LTCI in the first round of national pilot cities has reduced the number of hospitalizations, the total inpatient expenditure, and the out-of-pocket (OOP) inpatient expenditure among ineligible disabled individuals. The effects of LTCI on the hospital expenditures of ineligible disabled individuals are larger among middle-aged or urban groups, and are concentrated in schemes with coverage only of urban employee basic medical insurance, larger beneficiary population, higher reimbursement ceilings, and benefits only in kind. All three mechanisms, including output effect, health effect, and the substitution effect of formal care, are verified, while the substitution effect of informal care remains unclear.

**Conclusion:**

This study provides both theoretical and empirical evidence for the stepwise expansion and nationwide coverage of LTCI in China. The findings may also have policy implications for the establishment and development of long-term care (LTC) systems in other middle-income and developing countries confronted with increasing demand for LTC.

## Introduction

As one of the fastest aging countries in the world, China is experiencing a growing disabled population, which has created a widening gap between the demand for long-term care (LTC) and the available supply, and also an ever-rising medical expenditure. Furthermore, the inflation of medical expenses is exacerbating as disabled individuals, who are actually in need of LTC, inefficiently use medical services, especially hospital care. To better meet the LTC needs of disabled individuals, effectively control the rising medical expenses, and achieve a more efficient allocation of medical resources, the Chinese government declared to pilot long-term care insurance (LTCI) in 15 cities in June 2016. Meanwhile, as key pilot provinces, Jilin and Shandong could choose other cities within the province to pilot LTCI. Given this context, it is crucial to assess whether LTCI can reduce the medical expenditures for disabled individuals and achieve its goal of cost control.

The existing literature has not reached a consensus conclusion on the effects of LTC systems on medical expenses. The majority indicate that LTCI has reduced hospital utilization and hospital expenditures. In the US, Home- and Community-Based Services supplied by Medicaid are significantly correlated with a lower risk of hospitalization, though the effectiveness of some services diminishes over time (1). In Korea, the number of hospitalizations and the length of stay for LTCI beneficiaries have significantly reduced (2). In Spain, reforms to the LTC system have expanded public subsidization for LTC, which significantly reduced the hospital utilization among beneficiaries, including those receiving caregiving allowances and those opting for publicly funded home care, leading to an overall cost reduction of 11% (3). The LTCI pilot in China has significantly decreased outpatient and inpatient expenditures among the middle-aged and older adult population (4–6). However, McKnight (7) finds that the reform imposing strict average per-patient reimbursement caps in the US Medicare program has resulted in a significant decrease in the supply of home care services but has no impact on medical expenditures. Furthermore, Yu et al. (8) observed that after the implementation of LTCI in Qingdao, per capita medical expenses quickly rebounded and had been growing continually, following a brief decline. In summary, the existing literature has focused on beneficiaries or the whole population, leaving the effects of LTCI on medical expenditures among ineligible individuals unclear, theoretically and empirically.

Based on Becker’s household production function, we construct a theoretical framework to analyze the impacts of LTCI on medical expenditures among eligible and ineligible individuals. Disabled individuals, compared to their non-disabled counterparts, have an additional demand for a specific commodity: health related to their activities of daily living (HA), which should be produced in the household production with the investments of not only care services (such as LTC and hospital care) but also the non-market time of households. For the eligible, LTCI will directly lower the real price of LTC. Furthermore, it will reduce the non-market time needed to produce one unit of HA by increasing the supply of formal LTC services. Both changes will influence the household production process of HA, thereby generating substitution and output effects on its input factors, including hospital care. The substitution effect will reduce reliance on hospital care in household production, lowering hospital expenditures. Conversely, the output effect will increase the input of hospital care, resulting in higher hospital expenditures. In addition, LTCI can also enhance the production and consumption of HA, thereby improving the average HA level and overall health and generating a health effect. The health effect will reduce the input of hospital care and hospital expenditures. For the ineligible individuals, despite not receiving LTCI benefits and the real price of LTC remaining unchanged, LTCI will still reduce the non-market time required to produce one unit of HA by increasing the supply of formal LTC services. Consequently, it will also influence the utilization of hospital care and hospital expenditures among ineligible individuals through substitution, output, and health effects.

According to theoretical analysis, we examine the effects of LTCI on the hospital expenditures of ineligible individuals and then test the mechanisms through which LTCI exerts its effects, using the unbalanced panel data combined with four waves’ survey data of CHARLS in 2011, 2013, 2015, and 2018, and the corresponding years’ statistical data of China Urban Statistical Yearbook. First, we carefully identify ineligible individuals based on the dependency assessment scales, eligibility criteria, and the covered public medical insurance schemes of LTCI in the first round of pilot cities. Then, we apply a DID approach to examine the causal effects of LTCI on the hospital expenditures of ineligible disabled individuals. We demonstrate that the implementation of LTCI in the first national pilot cities in China has led to a decrease in the number of hospitalizations, the total inpatient expenditure, and the OOP inpatient expenditure among ineligible disabled individuals, but the effect on the probability of being hospitalized remains ambiguous. The impacts appear to be stronger among middle-aged or urban groups, and have been concentrated in schemes with coverage only of urban employee basic medical insurance, larger beneficiary population, higher reimbursement ceilings, and benefits only in kind. The output effect, health effect, and the substitution effect of formal care are verified, while the substitution effect of informal care remains unclear.

This study makes several contributions to the literature. Firstly, we provide a new theoretical explanation for the effects of LTCI on medical expenses among both eligible and ineligible individuals, which has not been examined theoretically to the best of our knowledge. Secondly, we provide empirical evidence demonstrating the significant negative effects of LTCI on medical expenditures among ineligible individuals. Thirdly, we verify the mechanisms through which LTCI influences the hospital expenditures of ineligible individuals. Finally, our findings regarding the indirect effects of LTCI on the hospital expenditures of ineligible individuals add new theoretical insights and empirical evidence for the indirect effects of government public policies on ineligible groups.

## Theoretical framework

### Theoretical model

According to the household production function proposed by Becker (9), fundamental human needs are not market goods, but commodities such as sleep and health. These commodities directly contribute to consumers’ utility and should be included in the utility function directly. However, consumers cannot simply purchase these commodities in the market. Instead, they should produce them through the productive activities of households, combining the purchased market goods and services and their own non-market time. Therefore, consumers’ demand for market goods is their induced demand for commodities. In this framework, the demand for LTC services is induced from the demand for HA, the unique health demand related to the activities of daily living of disabled individuals. Consequently, it should be produced through the productive activities of households, combining LTC and the non-market time of household members.

We consider a simple model of a representative household with a disabled individual to analyze how the implementation of LTCI affects hospital expenditures among eligible and ineligible disabled individuals. In this representative household, the disabled individual is a recipient of LTC, and the other household members are healthy, offering care themselves or assisting the disabled individual in searching for, purchasing, and utilizing formal LTC. All members are altruistic and highly interdependent with each other, ensuring that their utility functions are aligned. The household derives utility from various commodities, expressed through the following utility function:


(1) U=U(Z,HA)

Where *HA* stands for activities of daily living, an essential aspect of health, and *Z* represents all the other commodities required by the household except for *HA*.

The household produces commodities *Z* and *HA* according to the following household production functions:


(2) $$Z=G_{Z}(X,\mathrm{TX};E)$$

(3) $$\mathrm{HA}=G_{A}(M,\mathrm{TM},F,\mathrm{TF};E)$$

In Equations 2, 3, where *X* denotes a vector of market goods and services, *M* is a vector of inpatient medical care, namely the inpatient disease treatment and nursing care provided by comprehensive or specialized hospitals, *F* represents LTC, representing institutional care and hospital care provided by designated care service institutions such as hospitals which integrated eldercare services with medical care, nursing homes, and community nursing centers, or home care offered by relatives, neighbor, and friends, etc. Accordingly, *TX*, *TM*, and *TF* are the non-market time invested by the household in producing corresponding commodities. Finally, *E* represents the environmental variables that influence household production, reflecting the technology level of the household production process.

The household production faces the dual constraints of income and time:


(4) $$I=P_{X}X+P_{M}M+P_{F}F=\mathrm{wTW}+V$$

(5) T=TX+TM+TF+TW

Where *P_X_*, *P*_*M*,_ and *P_F_* are the prices of *X*、*M*, and *F*, respectively, *W* is the income earned per unit of working time, *TW* is the working time, *V* represents nonwage income, and *T* represents the total disposable time. Furthermore, we can collapse Equations 4, 5 into a single full income constraint, as specified in Equation 6:


(6) $$S=\mathrm{wT}+V=(\mathrm{wTX}+P_{X}X)+(\mathrm{wTM}+P_{M}M)+(\mathrm{wTF}+P_{F}F)$$

The household aims to maximize the utility given by Equation 1 subject to the constraints of the household production functions (2) and (3), as well as the full income constraint (6). The Lagrangian can be expressed by the following Equation 7:


(7) $$\begin{array}{l} L=U(Z,\mathrm{HA})-\\ \lambda [(\mathrm{wTX}+P_{X}X)+(\mathrm{wTM}+P_{M}M)+(\mathrm{wTF}+P_{F}F)-S] \end{array}$$

First-order optimality conditions for the commodities are:


(8) $$\frac{{\mathrm{MU}}_{Z}}{MU_{\mathrm{HA}}}=\frac{w\frac{\mathrm{dTX}}{\mathrm{dZ}}+P_{X}\frac{\mathrm{dX}}{\mathrm{dZ}}}{\left(w\frac{\mathrm{dTM}}{\mathrm{dHA}}+P_{M}\frac{\mathrm{dM}}{\mathrm{dHA}})+(w\frac{\mathrm{dTF}}{\mathrm{dHA}}+P_{F}\frac{\mathrm{dF}}{\mathrm{dHA}}\right)}\equiv \frac{\pi_{Z}}{\pi_{\mathrm{HA}}}$$

Where *MU_Z_* and *MU_HA_* are the marginal utilities of the commodities *Z* and *HA*, $\frac{\mathrm{dTX}}{\mathrm{dZ}}$ , $\frac{\mathrm{dX}}{\mathrm{dZ}}$ , $\frac{\mathrm{dM}}{\mathrm{dHA}}\;$ , $\frac{\mathrm{dF}}{\mathrm{dHA}}$ , $\frac{\mathrm{dTM}}{\mathrm{dHA}}$ , and $\frac{\mathrm{dTF}}{\mathrm{dHA}}$ represent the productivity of each input in producing *Z* or *HA*, while $\pi_{Z}$ and $\;\pi_{\mathrm{HA}}$ are shadow prices. In equilibrium, the ratio of the marginal utilities brought by *Z* and *HA* must equal the ratio of their respective shadow prices.

Similarly, the first-order conditions for the optimal inputs of all factors in household production are:


(9) $$\frac{\frac{\partial U}{\partial Z}\;\frac{\partial Z}{\partial f_{\mathrm{zk}}}}{\frac{\partial U}{\partial \mathrm{HA}}\;\frac{\partial \mathrm{HA}}{\partial f_{\mathrm{Al}}}}\equiv \frac{{\mathrm{MU}}_{Z}\;MP_{\mathrm{fzk}}}{MU_{\mathrm{HA}}\;MP_{\mathrm{fAl}}}=\frac{P_{f_{\mathrm{zk}}}}{P_{f_{\mathrm{Al}}}}\;$$

Where $f_{\mathrm{zk}}$ is the factor *k* put into the household production of commodity *Z*, and $f_{\mathrm{Al}}$ is the factor *l* used in producing commodity *HA*. $\frac{\partial U}{\partial Z}\frac{\partial Z}{\partial f_{\mathrm{Zk}}}$ and $\frac{\partial U}{\partial \mathrm{HA}}\frac{\partial \mathrm{HA}}{\partial f_{\mathrm{Al}}}$ represent the increased utilities brought by one unit increase in the input of production factor *k* and *l*, that is, the marginal utility of the production factor. In equilibrium, the ratio of the marginal utilities brought by two factors must equal the ratio of their respective prices. In particular, if both factors are put into the production of one commodity, for example, considering only *M* and *F* in producing *HA*, the equilibrium condition 9 will reduce to:


(10) $$\frac{\frac{\partial U}{\partial \mathrm{HA}}\;\frac{\partial \mathrm{HA}}{\partial M}}{\frac{\partial U}{\partial \mathrm{HA}}\;\frac{\partial \mathrm{HA}}{\partial F}}=\frac{MU_{\mathrm{HA}}{\mathrm{MP}}_{M}}{MU_{\mathrm{HA}}{\mathrm{MP}}_{F}}=\frac{{\mathrm{MP}}_{M}}{{\mathrm{MP}}_{F}}=\frac{P_{M}}{P_{F}}\;$$

And to determine the optimal inputs of *M* and *TF* in the production of *HA*, the Equation 9 becomes:


(11) $$\frac{\frac{\partial U}{\partial \mathrm{HA}}\;\frac{\partial \mathrm{HA}}{\partial M}}{\frac{\partial U}{\partial \mathrm{HA}}\;\frac{\partial \mathrm{HA}}{\partial \mathrm{TF}}}=\frac{MU_{\mathrm{HA}}{\mathrm{MP}}_{M}}{MU_{\mathrm{HA}}MP_{\mathrm{TF}}}=\frac{{\mathrm{MP}}_{M}}{MP_{\mathrm{TF}}}=\frac{P_{M}}{w}$$

In conclusion, the demands for inpatient medical care and LTC of disabled individuals are derivative demands for *HA*. Therefore, the implementation of LTCI will not only directly affect the inputs of factors during the household production process of *HA* but also indirectly influence these inputs by changing the consumption of *HA* in the consumption process of commodities.

### The impacts of LTCI on the hospital utilization of eligible individuals

In the pilot cities, LTCI primarily provides benefits by reimbursing the LTC expenses incurred by disabled individuals in designated institutions or subsidizing family caregivers with caregiving allowances. Therefore, the real price of LTC decreases consequently to $\;(1-\beta )P_{F}$ when the reimbursement or subsidy ratio is *β*. Moreover, the implementation of LTCI has the potential to improve the productivity of TF. Before the implementation of LTCI, households often spent a lot of time searching for, waiting for, and transporting to LTC services due to the supply–demand contradiction. This situation has been changing following the implementation of LTCI. Both the government and society have been increasing their investments in formal care services, leading to a significant rise in the availability of these services, particularly community- and home-based care (10). Consequently, the time households spend searching for, waiting for, and transporting to formal care services has decreased. This reduction in time means that the amount of time required for households to produce one unit of HA declines, resulting in a decrease in $\frac{\mathrm{dTF}}{\mathrm{dHA}}$ and an increase in $MP_{\mathrm{TF}}$ .

Firstly, when considering only the impacts of LTCI on the production process of *HA*, the implementation of LTCI will reduce $P_{F}$ to $(1-\beta )P_{F}$ and improve $MP_{\mathrm{TF}}$ to $MP_{\mathrm{TF}}^{\prime }\;$ ( $MP_{\mathrm{TF}}^{\prime }>MP_{\mathrm{TF}}$ ), thus changing Equations 10, 11 to $\frac{{\mathrm{MP}}_{M}}{{\mathrm{MP}}_{F}}<\frac{P_{M}}{(1-\beta )P_{F}}$ and $\frac{{\mathrm{MP}}_{M}}{MP_{\mathrm{TF}}^{\prime }}<\frac{P_{M}}{w}$ , respectively. Ceteris paribus, households will allocate more *F* and *TF*, meaning they will invest more LTC and related non-market time in the household production of *HA* until the ratio of marginal production (*M* and *F*, or *M* and *TF*) equals the ratio of their prices.

Secondly, when further considering the effects of LTCI on the consumption of *HA* and the subsequent impacts on its production process, there will be two changes. On the one hand, both the decrease of $P_{F}$ and the increase of $MP_{\mathrm{TF}}$ will lower the shadow price $\pi_{\mathrm{HA}}$ , changing Equation 8 to $\frac{{\mathrm{MU}}_{Z}}{MU_{\mathrm{HA}}}<\frac{\pi_{Z}}{\pi_{\mathrm{HA}}}$ , and thus enable households to consume more *HA*. On the other hand, the price index of all commodities π declines due to the decrease of $\pi_{\mathrm{HA}}$ , leading to an increase in real full income S/π , enabling households to increase their demands for *HA* (as well as all other normal commodities). In summary, LTCI will increase the demand for and consumption of *HA*, resulting in more inputs of all the factors in its production process, including hospital care.

**Figure 1:**
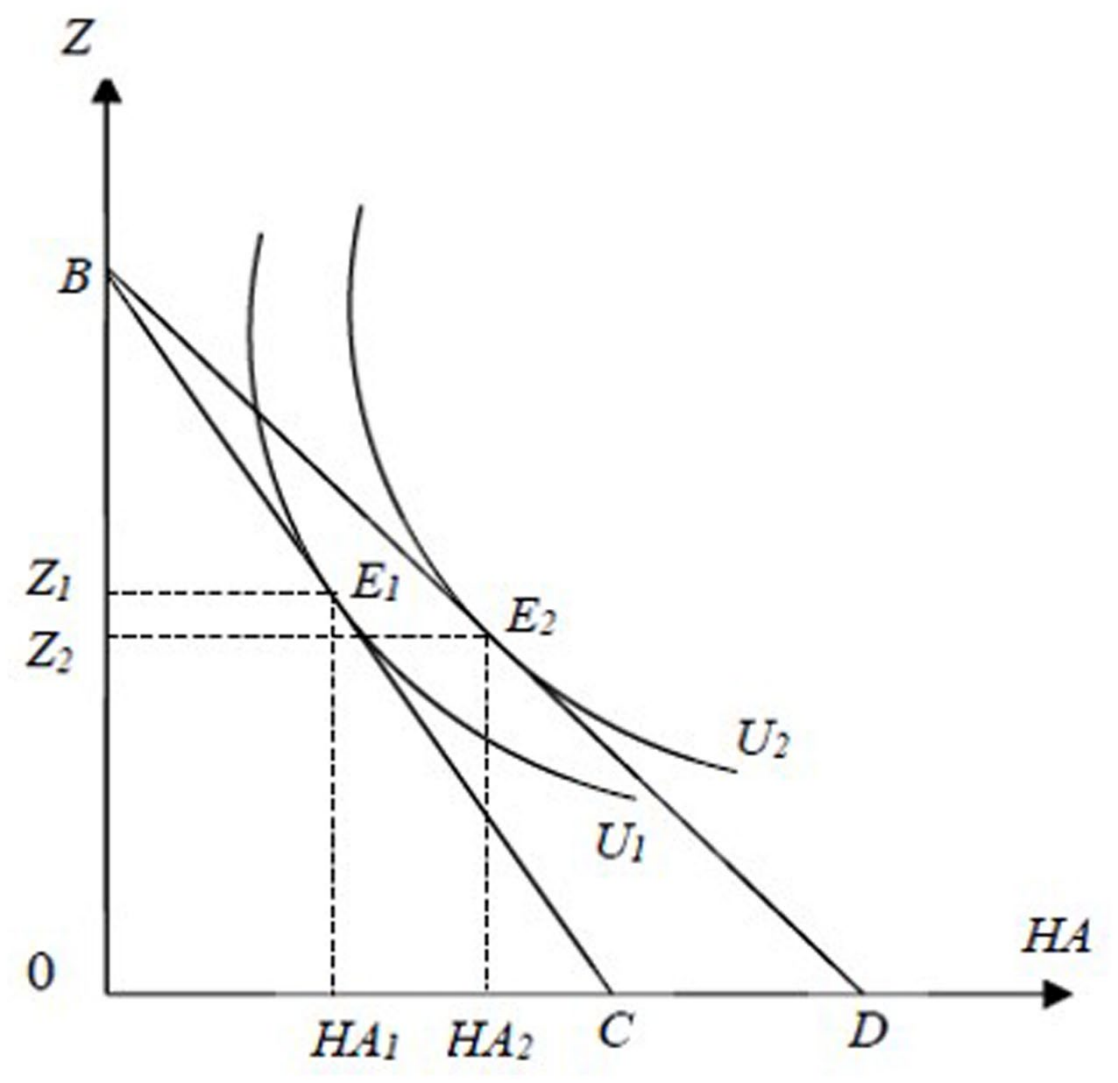
The effects of LTCI on the consumer equilibrium of commodities among disabled individuals. The *X*-axis represents commodity *HA* and the *Y*-axis indicates commodity Z. *U_1_* and *U_2_* are indifference curves while *BC* and *BD* are budget lines.

**Figure 2:**
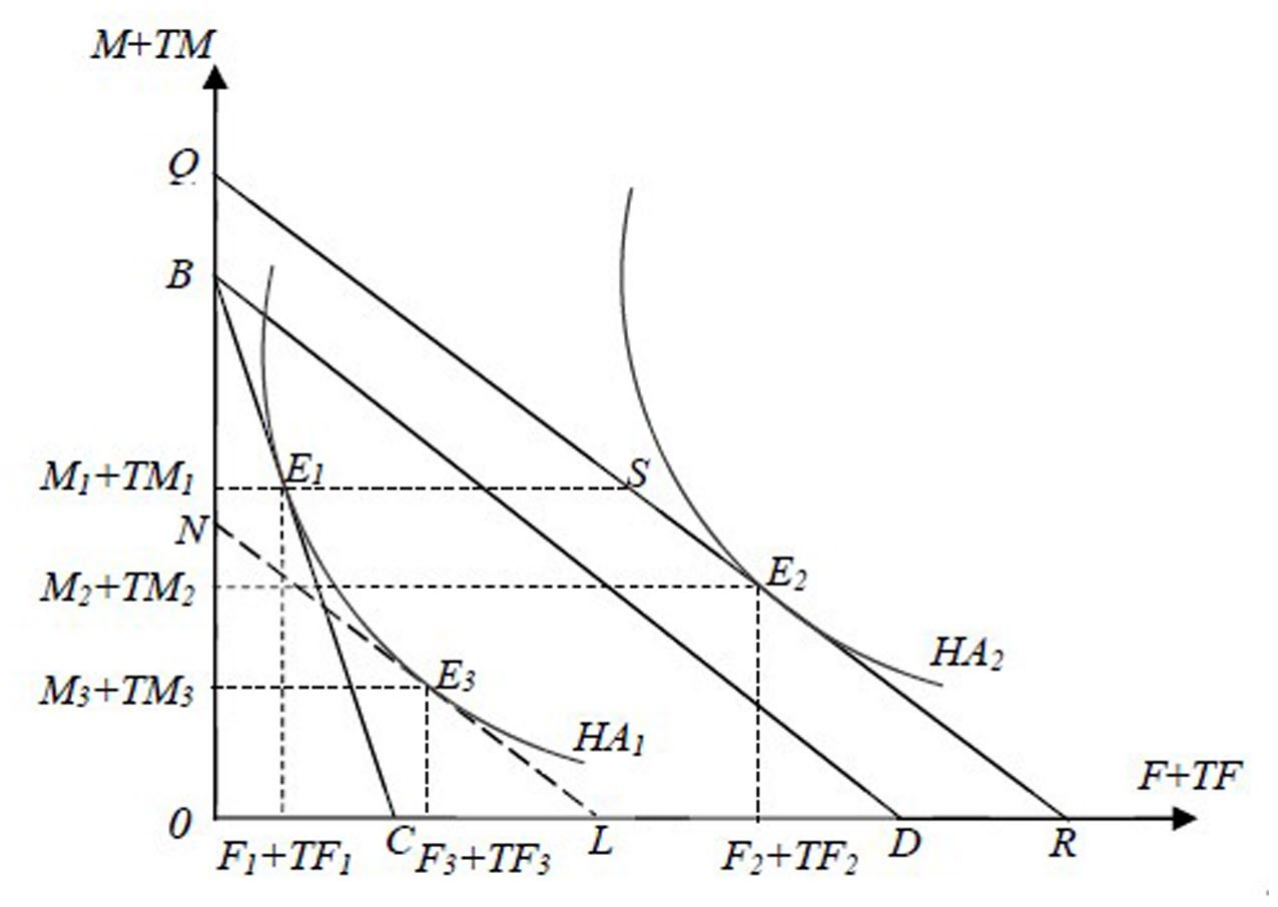
The effects of LTCI on the producer equilibrium of HA among disabled individuals. The *X*-axis represents *F* and *TF*,and the *Y*-axis indicates *M* and *TM* in the household production of *HA*. *HA_1_* and *HA_2_* are isoquants, while *BC*, *BD*, and *QR* are iso-cost lines.

Figures 1, 2 show the impacts of LTCI on hospital utilization among eligible individuals. Before the implementation of LTCI, in Figure 1, the equilibrium point of commodities for the representative household is *E_1_*, where the isoquant *U_1_* intersects with the budget line *BC*, and the corresponding consumption of *HA* is *HA_1_*. In Figure 2, to produce *HA_1_*, the required inputs for inpatient care and LTC are *M_1_* and *F_1_*, respectively. After LTCI is implemented, when considering only its impacts on the production process of *HA*, in Figure 2, the iso-cost line *BC* rotates outward to *BD*. When further considering the effects of LTCI on the consumption of *HA* and the subsequent impacts on its production process, in Figure 1, the budget line *BC* rotates outward to *BD*, which is tangent to the new isoquant line *U_2_* at the new equilibrium point *E_2_*, leading to an increase in *HA* consumption from *HA_1_* to *HA_2_*. This shift raises the iso-cost line from *BD* to *QR* in Figure 2. The new iso-cost line *QR* and the isoquant line *HA_2_* are tangent at the new equilibrium point *E_2_* (Note that the equilibrium point *E_2_* may fall at any position on the line segment QR). In summary, the direct impacts of LTCI on LTC and hospital care for eligible individuals can be divided into the substitution effect and output effect.

The substitution effect will enable disabled individuals to replace inpatient medical care with relatively lower-priced LTC in the production of *HA*, thereby increasing the inputs of LTC from *F_1_* to *F_3_* and reducing the investments in hospital care from *M_1_* to *M_3_*, maintaining *HA_1_* unchanged.

The output effect will increase *HA_1_* to *HA_2_*, thereby increasing the inputs of both LTC and hospital care, from *F_3_* to *F_2_* and *M_3_* to *M_2_*, respectively, due to both the decrease in real costs in the production of *HA* and its increased consumption, under the condition that the relative price of LTC and hospital care remains unchanged.

In addition, LTCI also has a health effect, which indirectly reduces the inputs of both LTC and hospital care in the production process of *HA* by improving the overall health level. As discussed above, LTCI will increase *HA_1_* to *HA_2_* in both the consumption and production processes of *HA*, indicating an improvement in the ability to perform daily activities. Furthermore, different dimensions of personal health are not independent of each other but interdependent intricately (11, 12). Psychological distress can manifest through physical symptoms such as pain, fatigue, and nausea (13–15). Older adult patients experiencing pain are more likely to suffer from depression and self-report as unhealthy (15). Consequently, LTCI will also enhance various health outcomes, including psychological and other dimensions of physiological health, thereby boosting their overall health level and generating a health effect.

### The productivity improvement of TF and the indirect impacts of LTCI on ineligible individuals

In China, LTCI is still in its early stages, so its coverage of the system is limited. Some disabled individuals, especially most of those who only have difficulties in instrumental activities of daily living (IADLs), do not qualify for the benefits. However, they may still benefit from LTCI if the productivity of *TF* has improved. Since LTCI will increase the supply of formal LTC services, especially community- and home-based care, and all designated institutions for LTCI can also provide services to the ineligible, the time spent searching, waiting, and transporting will drop subsequently. As a result, the needed nonmarket time *TF* for the ineligible to produce one unit of *HA* decreases, meaning the productivity of *TF* improves.

For ineligible disabled individuals, firstly, when considering only the impacts of LTCI on the production process of *HA*, the implementation of LTCI will improve $MP_{\mathrm{TF}}$ to $MP_{\mathrm{TF}}^{\prime }\;$ ( $MP_{\mathrm{TF}}^{\prime }>MP_{\mathrm{TF}}$ ), leading to $\frac{{\mathrm{MP}}_{M}}{MP_{\mathrm{TF}}^{\prime }}<\frac{P_{M}}{w}$ . Ceteris paribus, households will use more *TF* in the household production of *HA*, and the use of LTC will increase accordingly for the complementary relationship of the two factors *TF* and *F*. Secondly, when further considering the effects of LTCI on the consumption of *HA* and the resulting impacts on its production process, as $\frac{\mathrm{dTF}}{\mathrm{dHA}}$ decreases, $\pi_{\mathrm{HA}}$ drops, changing Equation 8 to $\frac{{\mathrm{MU}}_{Z}}{MU_{\mathrm{HA}}}<\frac{\pi_{Z}}{\pi_{\mathrm{HA}}}$ . In addition, as $\pi_{\mathrm{HA}}$ drops, π falls, S/π rises, and the demand for and consumption of *HA* increase subsequently, resulting in increased inputs of all factors in its production process, including hospital care. Therefore, LTCI can also affect the hospital expenditures of ineligible disabled individuals through substitution, output, and health effects.

There may be concern that the effect of LTCI may be too modest to affect ineligible individuals. However, if the relative effects of income and time constraints are involved, for households with lower income and higher time constraints, there could be significant effects. Such households are not uncommon in China nowadays. For instance, a survey conducted in the Xincheng District of Beijing shows that 45% of older adult care services are provided through hiring nannies (16), indicating that these households primarily face time constraints rather than income constraints when selecting care options. Although the urban areas of Beijing cannot represent the general situation nationwide, it is worth noting that the survey was conducted from 2004 to 2005, and the income level of Chinese residents has considerably improved since then, with nearly 20 years of rapid development. Between 2005 and 2022, the per capita income of urban residents in China increased 3.7 times, reaching 49282.9 yuan, while the per capita net income of rural residents grew 5.2 times to 20132.8 yuan. Moreover, all the first national pilots of LTCI are cities with higher economic strength and income levels within each province and even the whole country. Given these factors, we can expect significant impacts of LTCI on ineligible individuals.

**Figure 3:**
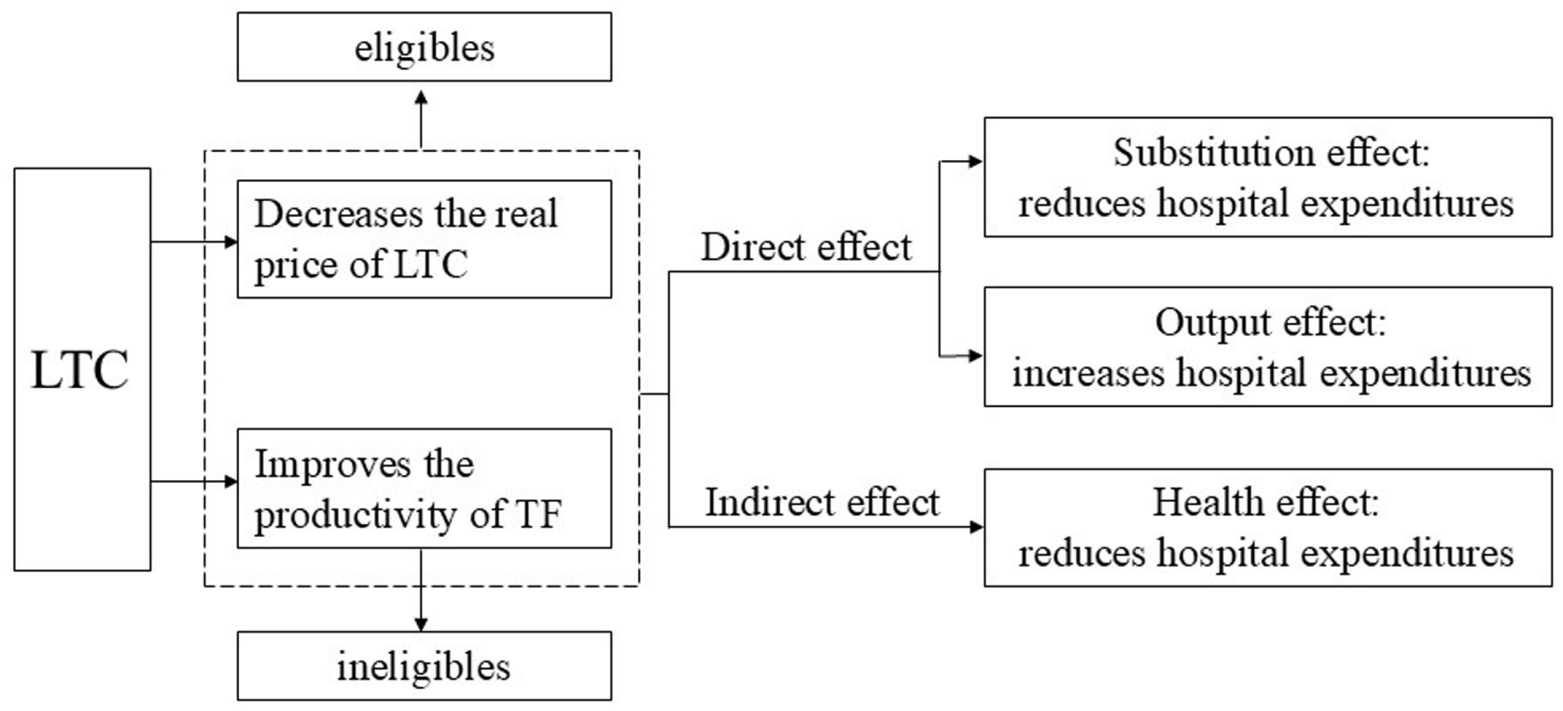
The effects of LTCI on hospital expenditures.

In summary, the effects of LTCI on hospital expenditures can be expressed as shown in Figure 3. For the eligible, LTCI not only lowers the real price of LTC but also enhances the productivity of TF, thereby influencing their hospital expenditures through a negative substitution effect, a positive output effect, and a negative health effect. For the ineligible, LTCI can also improve the productivity of TF, thereby influencing their hospital expenditures through substitution, output, and health effects.

## Data and variables

### Data source and study sample

The data are from the CHARLS and China City Statistical Yearbook. The individual data are sourced from the four waves of survey data conducted by CHARLS in 2011, 2013, 2015, and 2018. CHARLS is a longitudinal survey jointly executed by Wuhan University and Peking University, collecting abundant information from a nationally representative sample of Chinese residents aged 45 or older. The individual information on physical dysfunction, medical expenses, demographics, and socio-economic status in CHARLS is used in this study. The statistical data at the city level come from the 2012, 2014, 2016, and 2019 editions of the China City Statistical Yearbook, which include primary statistical data on the socio-economic development of over 650 cities across the country. The city-level information on population, economy, and health in the yearbooks is used in this study.

This study concentrates on the indirect effects of LTCI on ineligible disabled individuals. As introduced above, we identify ineligible individuals based on the dependency assessment scales, eligibility criteria, and the covered public medical insurance schemes in the first round of pilot cities (see Supplementary Table S1). Therefore, we first identified 8,568 disabled individuals who reported having difficulties in performing activities of daily living (ADLs) and IADLs. Second, we made more sample restrictions: excluding respondents from Shandong and Jilin provinces to address endogeneity brought about by the self-selection bias of implementation date within the two key pilot provinces; deleting respondents from Chongqing because LTCI in this city implemented in May 2019 instead of December 2017, when its pilot program was released; eliminating respondents from the four autonomous pilot cities, namely Xuzhou in Jiangsu Province, Hangzhou and Jiaxing in Zhejiang Province, and Linfeng in Shanxi Province, to avoid their interference; dropping respondents from Shanghai, because the dependency assessment scale there include some items of IADLs and LTCI there covers individuals with mild disability, enabling those only dependent in IADLs may be eligible for LTCI benefits, a situation not found in other pilot cities. Consequently, without the coverage of Changchun, Shihezi, and Nantong in CHARLS, only 9 national pilot cities remain in our treatment group. Third, we eliminate respondents who enrolled in urban employee basic medical insurance (UEBMI) in Chengde, Qiqihaer, Ningbo, Anqing, Shangrao, Guangzhou, and Chengdu, and drop those in UEBMI or urban–rural resident basic medical insurance (URRBMI) in Suzhou and Jingmen. After deleting all observations with missing data, the final sample includes 13,156 observations of 5,857 respondents.

### Variables

We select four outcome variables to measure hospital expenditures: hospital admission, number of hospitalizations, total inpatient expenditure, and OOP inpatient expenditure. Hospital admission is a binary variable equal to 0 if the respondent did not receive inpatient care over the past year and equal to 1 if they had. Respondents who had received inpatient care in the past year further reported the number of hospitalizations. Therefore, this variable is a count, taking a positive number for those who had received inpatient care at least once, and 0 for those who had not. Total inpatient expenditure comes from the question, “What was the total medical cost for all the inpatient care you received during the past year?” OOP inpatient expenditure derives from the question, “How much is the OOP part?”

The key independent variable is whether the city in which the disabled individual lived had implemented LTCI, which is determined by two factors. The first is whether the disabled individual lived in one of the 9 national pilot cities. If so, they are classified into the treatment group, while others fall into the control group. The other factor is the year. In this study, all 9 national pilot cities implemented LTCI in 2016 or 2017, so 2018 was post-pilot, and 2011, 2013, and 2015 were pre-pilot.

## Empirical strategies

A natural experiment occurred when LTCI was implemented in the first round of pilot cities. There are two variations in medical expenditures: between pilot and non-pilot, and between the periods before and after the rollout of LTCI. Moreover, as the 9 national pilot cities carried out LTCI in 2016 and 2017, the four waves of data in 2011, 2013, 2015, and 2018 provide just three waves before and one wave after the official implementation of LTCI. Consequently, the standard DID is applied to identify the causal effects, as indicated in the following equation:


(12) $$Y_{\mathrm{ict}}=\beta_{0}+\beta_{1}\text{Trea}t_{\mathrm{ic}}\times {\text{Post}}_{t}+\beta_{2}X_{\mathrm{ict}}+\beta_{3}W_{\mathrm{ct}}+\tau_{t}+\alpha_{i}+\varepsilon_{\mathrm{ict}}$$

The dependent variable, $Y_{\mathrm{ict}}$ , represents the hospital expenditures of a disabled individual *i* living in city *c* in year *t*, including hospital admission, number of hospitalizations, total inpatient expenditure, and OOP inpatient expenditure. The key independent variable is the interaction term $\text{Trea}t_{\mathrm{ic}}\times {\text{Post}}_{t}$ , where $\text{Trea}t_{\mathrm{ic}}$ takes the value 1 if the disabled individual *i* lived in one of the 9 national pilot cities and 0 if they did not, and ${\text{Post}}_{t}$ is equal to 1 for the post-pilot year 2018 and 0 for the three pre-pilot years 2011, 2013, and 2015. *X_ict_* refers to time-variant individual characteristics, including age, marital status, education, urban residence, number of living children, UEBMI, and URRBMI, and $W_{\mathrm{ct}}$ denotes time-variant city-level characteristics, namely natural growth rate of population, GDP per capita, fiscal expenditure per capita, number of hospital beds per 1,000 inhabitants, and number of doctors per 1,000 inhabitants. $\tau_{t}$ , $\alpha_{i}$ , and $\varepsilon_{\mathrm{ict}}$ represent time FE, individual FE, and the random error term, respectively. The coefficient of $\text{Trea}t_{\mathrm{ic}}\times {\text{Post}}_{t}$ , $\beta_{1}$ , captures the impacts of LTCI averaging across all disabled individuals who are ineligible for benefits. Standard errors are clustered at the city level.

The primary concern of our identification strategy is the selection bias from the non-randomness in the selection of pilot cities. Factors at the city level, such as economic and financial strengths, population aging, and medical and health conditions, may not only affect the central government’s selection of pilot cities but also influence the urgency of local governments to implement LTCI. Meanwhile, these factors may be associated with the medical expenses of residents. Economic growth and overall economic strength may relate to the income levels of residents, thereby affecting their demand for medical services and the corresponding medical expenses. Population aging may affect medical costs by increasing the overall demand for healthcare services. To address this concern, we control for two-way FE and five city-level variables, as specified in Equation 12, to alleviate biases from all time-invariant observable, unobservable, and time-variant city-level factors, respectively. Furthermore, we test the robustness of the baseline estimates by selecting only the second round of national pilot cities as the control group. As the second round of national pilot cities 4 years later, these cities may be more similar to the first ones (17), thus minimizing the differences between the treatment and control groups.

Another selection bias may arise if people migrate from non-pilot to pilot cities or shift from uncovered public medical insurance to covered. As introduced in Section Data and variables, LTCI programs in most pilot cities cover only urban employees and retirees enrolled in UEBMI, so disabled or high-risk individuals may be inclined to switch from URRBMI to UEBMI. However, these issues are unlikely to affect the results of this study. For one thing, UEBMI covers urban employees. It is hard for urban and rural disabled residents to shift from URRBMI to UEBMI. For another, the cost of migration may be too high to afford, especially for households with disabled individuals. Under the expectation of expanding LTCI coverage, they may have little desire to change their current situation. Nonetheless, we select propensity score matching difference in differences (PSM-DID) to test the robustness of the baseline estimates. We match disabled individuals in the treatment group with more similar control individuals through propensity score matching (PSM), and then estimate policy effects with the matched sample.

There may still be the potential threat of omitted variables. For example, factors related to city-level facilities for older adults, such as residential institutions and residential care beds, are not included due to data inaccessibility. In addition, without the information on population aging, we select the natural growth rate of population as the proxy variable. However, other factors, such as population density, population mobility, birth concept, etc., may be missed. To address these issues, we apply the method proposed by Oster (18) to assess the effect of omitted factors by adjusting the baseline estimates.

## Results

### Descriptive statistics

Table 1 shows descriptive statistics for the full sample, as well as for the treatment and control groups before and after the implementation of LTCI. The results of pairwise comparisons between the treatment and control groups before the LTCI pilot are also reported. For the full sample, 22.2% had received inpatient care in the past year, and the average number of hospitalizations was 0.370. The average total and OOP inpatient expenditures are 3,052 and 1,589 yuan, respectively. After the implementation of LTCI, the proportion and mean values of the four outcome variables all go up. Compared to the control group, the treatment group showed smaller increases in hospitalization admissions and number of hospitalizations, but larger increases in total and OOP inpatient expenditures. The treatment group has not performed as expected in controlling total and OOP inpatient expenditures. However, this issue may also derive from the unbalanced panel data used in this study. There are missing observations in each period, which leads to an imbalance in the number of samples involved in the calculation of means before and after the pilot, thereby affecting the means of the treatment and control groups. *T*-test results show significant differences between the treatment group and control group in three individual variables: marital status, number of living children, and UEBMI, as well as all five city-level variables before the LTCI pilot. The two groups were unbalanced before the pilot, which requires further addressing.

**Table 1:** Descriptive statistics.

Variables	Full		Before pilot		After pilot	
			Treated	Control	Treated	Control
	Mean/%	Standard deviation	Mean/%	Mean/%	Mean/%	Mean/%
Hospital admission = 1	0.222	0.416	0.183	0.198	0.218	0.266
Number of hospitalizations	0.370	0.891	0.255	0.319	0.343	0.467
Total inpatient expenditure	3,052	12,624	2,210	2,320	4,652	4,177
OOP inpatient expenditure	1,589	7,345	927.3	1,243	2,273	2,158
Age	65.76	10.39	64.67	64.78	66.80	67.38
Married = 1	0.750	0.433	0.733*	0.765	0.712	0.730
Education (Junior high school and above = 1)	0.155	0.362	0.148	0.153	0.148	0.159
Urban residence	0.152	0.359	0.174	0.154	0.143	0.148
Number of living children	3.176	1.546	3.026**	3.168	3.036	3.218
Has UEBMI = 1	0.0740	0.261	0.047**	0.072	0.039	0.084
Has URRBMI = 1	0.858	0.349	0.840	0.849	0.873	0.874
GDP per capita	42,923	27,513	56789***	37,007	73,762	48,122
Fiscal expenditure per capita	8,079	4,681	9383***	6,702	13,769	9,647
Number of hospital beds per 1,000 inhabitants	4.239	1.550	5.065***	4.093	5.732	4.248
Number of doctors per 1,000 inhabitants	2.041	0.929	2.490***	1.831	3.081	2.235
Natural growth rate of population	6.647	4.713	5.601***	7.059	5.618	6.201

The *t*-test is applied for pairwise comparisons between the treatment and control groups before the pilot of LTCI. ***, **, and * mean the significance levels of 1, 5, and 10%, respectively.

### Baseline estimates

Table 2 shows the estimated effects of LTCI on hospital admission, number of hospitalizations, total inpatient expenditure, and OOP inpatient expenditure. The coefficients of interaction Treat_ic_ × Post_t_ in columns 2 to 4 are all significant, demonstrating that the implementation of LTCI has indeed reduced the hospital expenditures of ineligible disabled people. In particular, LTCI has significantly reduced the number of hospitalizations by 0.069, the total inpatient expenditure by 41.4%, and the OOP inpatient expenditure by 31.7%. LTCI can not only help to alleviate the pressure on hospital medical resources, but also be profitable to reduce the financial burdens on medical insurance funds and families of disabled individuals. However, the estimate in column 1 is not statistically significant. There is no evidence to support the effect of LTCI on the probability of being hospitalized among ineligible disabled individuals.

**Table 2:** Effects of LTCI on the hospital expenditures of ineligible disabled individuals.

Variables	(1)	(2)	(3)	(4)
	Hospital admission	Number of hospitalizations	Ln (Total inpatient expenditure)	Ln (OOP inpatient expenditure)
Treat_ic_ × Post_t_	−0.0341	−0.0694*	−0.414**	−0.317*
	(0.0240)	(0.0409)	(0.208)	(0.188)
Individual covariates	Yes	Yes	Yes	Yes
City-level covariates	Yes	Yes	Yes	Yes
Individual FE	Yes	Yes	Yes	Yes
Year FE	Yes	Yes	Yes	Yes
Observations	13,156	13,156	12,789	12,789
R-squared	0.012	0.013	0.016	0.014

Standard errors clustered at the city level are reported in parentheses. ***, **, and * mean the significance levels of 1, 5, and 10%, respectively. Each regression controls for individual covariates, city-level covariates, individual FE, and year FE. Individual covariates include age, marital status, education, urban residence, number of living children, UEBMI, and URRBMI. City-level covariates include Ln (GDP per capita), Ln(fiscal expenditure per capita), number of hospital beds per 1,000 inhabitants, number of doctors per 1,000 inhabitants, and natural growth rate of population.

### Robustness

**Figure 4:**
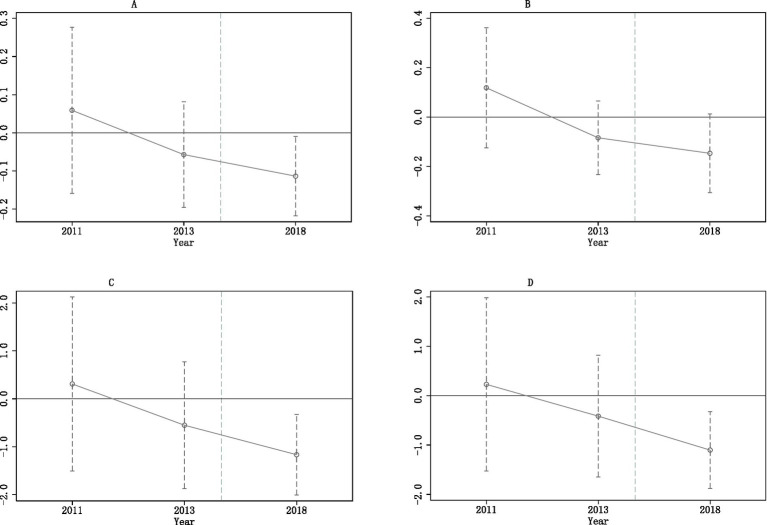
Parallel trend tests. The LTCI was implemented during 2016 and 2017 in the 9 pilot cities. On the x-axis, year 2015 is omitted because it is treated as the benchmark year. Each regression controls for individual covariates, city-level covariates, individual FE, and year FE. Individual and city-level covariates are the same as in Table 2. **(A)** Hospital admission. **(B)** Number of hospitalizations. **(C)** Total inpatient expenditure. **(D)** OOP inpatient expenditure.

First, we test the parallel trend assumption. The validity of our baseline estimates hinges on the test results of the parallel trend assumption, the key assumption of DID. It requires that the potential trends of hospital expenditures are parallel to each other had LTCI not been implemented, conditional on the covariates of Equation 12. The event-study specification is applied to test the assumption, and the results are shown in Figure 4. In all four panels in Figure 4, the horizontal axis represents the year, while the vertical axis denotes the estimated coefficient and its corresponding confidence interval. All estimates for 2011 and 2013 are not significant, indicating that compared to the benchmark year 2015, there were no significant differences between the treatment and control groups in hospital expenditures in 2011 or 2013. Thus, the trends of hospital expenditures in the treatment group are parallel to those in the control group before the implementation of LTCI. We can infer that the counterfactual trends of hospital expenditures in the treatment and control groups are parallel to each other. In addition, the estimates for 2018 in all four figures are significantly negative, including the one for hospital admission. Compared to the benchmark year 2015, the probability of being hospitalized, the number of hospitalizations, total inpatient expenditure, and OOP inpatient expenditure are all reduced significantly, indicating that LTCI may also have a significant impact on the probability of being hospitalized among ineligible disabled individuals.

Second, we replace the control group. We remain respondents from 7 national pilot cities of the second round, namely Beijing, Nanning, Hohhot, Tianjin, Kunming, Hanzhong, and Fuzhou, which CHARLS covers, as the control group. This adjustment reduced the total sample size to 1,543 observations, with 965 in the treatment group and 578 in the control group. The DID regression results are shown in Table 3. After the implementation of LTCI, the probability of being hospitalized for ineligible disabled individuals has reduced significantly by 11.8 percentage points, and the total and OOP inpatient expenditures have decreased by 111.2 and 86.7%, respectively. The number of hospitalizations has reduced by 0.129, though the estimate is only close to significant (*p* = 13.3). The coefficients here are much higher when compared to baseline estimates. It may be because, just as there was non-randomness in the selection of the first round of pilot cities, the second batch was not random either. Various factors, such as economic development, financial strength, health conditions, and the degree of population aging at the city level, may have affected the generation of the list. These cities are facing more pressure from the continuous growth of medical expenses, just like the first ones. Thus, a more similar control group results in higher estimated impacts of LTCI.

**Table 3:** Robustness test: replacing the control group.

Variables	(1)	(2)	(3)	(4)
	Hospital admission	Number of hospitalizations	Ln (Total inpatient expenditure)	Ln (OOP inpatient expenditure)
Treat_ic_ × Post_t_	−0.118*	−0.129	−1.112**	−0.867*
	(0.0556)	(0.0809)	(0.485)	(0.431)
Observations	1,543	1,543	1,506	1,506
R-squared	0.047	0.043	0.043	0.036

Standard errors clustered at the city level are reported in parentheses. ***, **, and * mean the significance levels of 1, 5, and 10%, respectively. Each regression controls for individual covariates, city-level covariates, individual FE, and year FE. Individual and city-level covariates are the same as in Table 2.

Third, we combine PSM with DID. To alleviate the significant differences in some observable variables between the control and treatment groups, PSM is applied to select a more similar control group on all the observables for the treatment group year by year. Then, we apply DID regression using the matched sample. Specifically, we select more similar individuals by the kernel matching method within 0.0001 calipers of propensity scores, which are estimated by logit models considering gender and all individual and city-level control variables in the baseline regression. The results of PSM-DID are shown in Table 4. LTCI has led to a reduction in hospital admission by 6.78 percentage points, a decrease in the number of hospitalizations by 0.142, a drop in the total inpatient expenditure by 71.4%, and a decline in the OOP inpatient expenditure by 52.7%. These results are slightly higher in magnitude than the baseline estimates, demonstrating that LTCI has reduced the hospital expenditures of ineligible disabled individuals.

**Table 4:** Robustness test: combining PSM with DID.

Variables	(1)	(2)	(3)	(4)
	Hospital admission	Number of hospitalizations	Ln (Total inpatient expenditure)	Ln (OOP inpatient expenditure)
Treat_ic_ × Post_t_	−0.0678*	−0.142*	−0.714**	−0.527*
	(0.0402)	(0.0775)	(0.309)	(0.269)
Observations	3,874	3,874	3,775	3,775
R-squared	0.024	0.029	0.031	0.035

Standard errors clustered at the city level are reported in parentheses. ***, **, and * mean the significance levels of 1, 5, and 10%, respectively. Each regression controls for individual covariates, city-level covariates, individual FE, and year FE. Individual and city-level covariates are the same as in Table 2.

Fourth, we assess the selection bias from omitted variables. The bias-adjusted coefficients are calculated based on the formula that Oster (18) obtained through random simulation. In particular, the results of baseline regressions are treated as the “full controls” specification, and those of DID regressions controlling for only two-way FE as the “restricted controls” specification. In addition, R_max_ is assumed to be 1.3R^2^, where R^2^ is the R-squared in the “full controls” specification, and the relative degree of selection on observables and unobservables *δ* is equal to 1. This robustness test is passed if the calculated adjusted coefficient is still within the 95% confidence interval of the coefficient of the key explanatory variable in the “full controls” specification. Table 5 reports the results of this adjustment method. While the results in the “full controls” specification are represented in column 1 and those in the “restricted controls” specification in column 2, the bias-adjusted coefficients are shown in column 3. All four coefficients in column 3 are still negative and quantitatively very close to the baseline estimates in the “full controls” specification, as well as within the corresponding 95% confidence interval in column 1, suggesting that the selection bias from omitted variables is too limited to change the conclusion of our baseline regressions.

**Table 5:** Robustness test: assessing selection bias from omitted variables.

Variables	(1)	(2)	(3)
	Full controls	Restricted controls	Bias adjusted coefficients
Panel A: Hospital admission			−0.032
Treat_ic_ × Post_t_	−0.0341	−0.0358	
Robust standard error	(0.0240)	(0.0247)	
95% confidence interval	[−0.0817, 0.0135]	[−0.0848, 0.0132]	
R-squared	0.012	0.008	
Panel B: Number of hospitalizations			−0.061
Treat_ic_ × Post_t_	−0.0694*	−0.0771*	
Robust standard error	(0.0409)	(0.0431)	
95% confidence interval	[−0.1506, 0.0119]	[−0.1626, 0.0084]	
R-squared	0.013	0.010	
Panel C: Ln (Total inpatient expenditure)			−0.450
Treat_ic_ × Post_t_	−0.414**	−0.387*	
Robust standard error	(0.208)	(0.204)	
95% confidence interval	[−0.8272, −0.0013]	[−0.7925, 0.0185]	
R-squared	0.016	0.012	
Panel D: Ln (OOP inpatient expenditure)			−0.342
Treat_ic_ × Post_t_	−0.317*	−0.298	
Robust standard error	(0.188)	(0.188)	
95% confidence interval	[−0.6897, 0.0550]	[−0.6703, 0.0746]	
R-squared	0.014	0.011	

The estimates with full controls are reported in column (1), and those with restricted controls are in column (2). The covariates in each regression with full controls include individual covariates, city-level covariates, individual FE, and year FE. Individual and city-level covariates are the same as in Table 2. The bias adjusted coefficients are in column (3). ***, **, and * mean the significance levels of 1, 5, and 10%, respectively.

We also apply the staggered DID and placebo test to verify the robustness of our baseline estimates. The former includes all respondents from the four autonomous pilot cities and the two key provinces. The latter changes the pilot year or generates the treatment group randomly. All the results confirm the robustness of our baseline estimates (see Supplementary Tables S2, S3; Supplementary Figure S1).

### Heterogeneity

**Figure 5:**
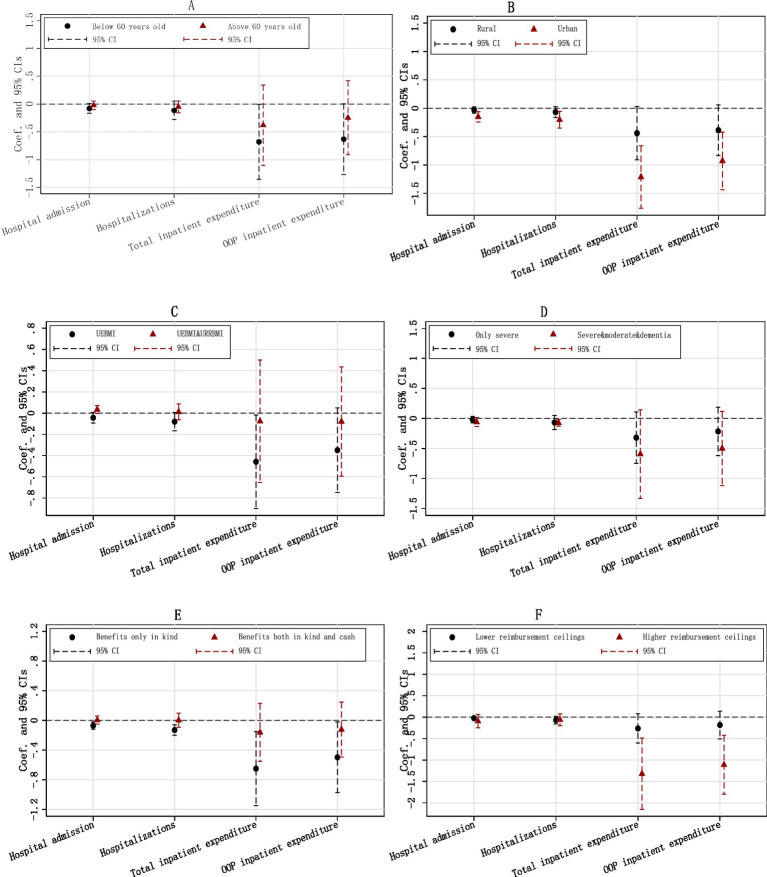
Heterogeneous effects. The Y-axis represents the estimated coefficients and their respective confidence intervals, while the X-axis indicates four outcomes of hospital expenditures. Each regression controls for individual covariates, city-level covariates, individual FE, and year FE. Individual and city-level covariates are the same as in Table 2. **(A)** By age. **(B)** By urban/rural residence. **(C)** By coverage. **(D)** By eligibility. **(E)** By benefit. **(F)** By reimbursement.

The impacts of LTCI on the hospital expenditures of ineligible disabled individuals may vary across various dimensions, such as different individual characteristics or policy designs. Therefore, we separate the whole sample into two subsamples based on each dummy grouping variable and regress Equation 12 using each subsample. Figure 5 shows the heterogeneous effects.

Figure 5A demonstrates that the significant effects of LTCI on hospital expenditures for the ineligible are primarily observed in those under 60 years of age. Among this younger subgroup, the three estimates concerning hospital admission, total hospital expenditure, and OOP hospital expenditure are all significant, while the one on the number of hospitalizations is not. In contrast, for individuals aged 60 and older, none of the four estimates is statistically significant.

Figure 5B illustrates that LTCI has effectively reduced the hospital expenditures for both urban and rural populations. However, while all four estimates for the urban sample are significant at the 1% level, only two of the four for the rural sample reach significance at the 10% level. Additionally, all the coefficients of Treat_ic_ × Post_t_ for the urban sample are much greater than those for the rural sample, indicating that the effects of LTCI on hospital expenditures are larger among the urban group.

Figures 5C–F display the heterogeneous effects across different policy designs. The impact of LTCI on hospital expenditures appears to be concentrated in schemes which cover only UEBMI, benefit a larger population, draw up higher reimbursement ceilings, or provide benefits only in kind.

### Mechanisms

We proceed to test the three mechanisms, namely substitution, output, and health effects, by estimating the impacts of LTCI on the following mechanism variables: formal caregiving, formal caregiving hours, family caregiving, family caregiving hours, severe disability in ADLs, severe disability in IADLs, self-reported health, and depression. However, this empirical strategy faces a problem: variations in LTC, including formal and informal care, are not caused solely by the substitution effect, but by a combination of substitution, output, and health effects. Therefore, we first estimate the average impacts of LTCI on the four variables, namely severe disability in ADLs, severe disability in IADLs, self-reported health, and depression, to verify the output effect and health effect. Then, following Nunn and Wantchekon (19), we estimate the average impacts of LTCI on proxy variables of the substitution effect, further controlling for the four proxy variables of output and health effects in each DID regression to verify the substitution effect.

Tables 6, 7 show the results of DID regressions estimating the impacts of LTCI on mechanism variables. In both tables, Panel A reports the estimates of LTCI with benefits only in kind, while Panel B represents those with benefits in kind and cash. Note that there will be no substitution effect of informal caregiving on hospital expenditures when LTCI provides only benefits in kind. On the contrary, formal LTC will exert a substitution effect on informal caregiving, which is not related to our topic, the mechanisms of LTCI on hospital expenditures. Therefore, estimates of LTCI on informal caregiving and informal caregiving hours are not reported in Table 7A when LTCI offers benefits only in kind.

**Table 6:** Output and health effects.

Variables	(1)	(2)	(3)	(4)
	Severe dependent in ADLs	Severe dependent in IADLs	Self-reported health	Depression
Panel A: Benefits only in kind				
Treat_ic_ × Post_t_	−0.0294***	−0.0275*	−0.0726***	−0.0525***
	(0.0108)	(0.0163)	(0.0256)	(0.0197)
Observations	12,755	12,755	11,595	11,182
R-squared	0.009	0.004	0.004	0.015
Panel B: Benefits in kind and cash				
Treat_ic_ × Post_t_	−0.0181**	−0.00237	−0.0130	−0.144**
	(0.00688)	(0.0231)	(0.0110)	(0.0631)
Observations	12,576	12,576	11,438	11,041
R-squared	0.009	0.004	0.003	0.015

Standard errors clustered at the city level are reported in parentheses. ***, **, and * mean the significance levels of 1, 5, and 10%, respectively. Each regression controls for individual covariates, city-level covariates, individual FE, and year FE. Individual and city-level covariates are the same as in Table 2.

**Table 7:** Substitution effect.

Variables	(1)	(2)	(3)	(4)
	Formal caregiving	Ln (Formal caregiving hours)	Informal caregiving	Ln (Informal caregiving hours)
Panel A: Benefits in kind				
Treat_ic_ × Post_t_	0.0478**	0.145**		
	(0.0191)	(0.0642)		
Two-way FE	Yes	Yes	Yes	Yes
Individual and city-level covariates	Yes	Yes	Yes	Yes
Output and health effects	Yes	Yes	Yes	Yes
Observations	11,166	8,862		
R-squared	0.010	0.006		
Panel A: Benefits in kind and cash				
Treat_ic_ × Post_t_	0.0426	0.0433**	−0.0224	0.144
	(0.0407)	(0.0188)	(0.0240)	(0.234)
Two-way FE	Yes	Yes	Yes	Yes
Individual and city-level covariates	Yes	Yes	Yes	Yes
Output and health effects	Yes	Yes	Yes	Yes
Observations	11,027	8,734	11,027	8,736
R-squared	0.009	0.004	0.177	0.123

Standard errors clustered at the city level are reported in parentheses. ***, **, and * mean the significance levels of 1, 5, and 10%, respectively. Each regression controls for individual covariates, city-level covariates, output and health effects, individual FE, and year FE. Output and health effects include four variables: severe disability in ADLs, severe disability in IADLs, self-reported health, and depression. Individual and city-level covariates are the same as in Table 2.

In Table 6A, the four estimates are all significant, demonstrating that when providing benefits only in kind, LTCI has significantly reduced the probability of severe disability in ADLs, severe disability in IADLs, self-reported unhealthy, and depression. Both output and health effects are verified. In Table 7A, further controlling for the output and health effects, LTCI has still decreased the possibility of formal caregiving by 4.78 percentage points, and formal caregiving hours by 14.5%. The substitution effect is validated.

In Table 6B, only the two estimates in columns 1 and 4 are significant, indicating that when providing benefits both in kind and cash, LTCI has negative impacts on the probability of severe disability in ADLs and the likelihood of depression. The output and health effects are also verified, but may be relatively small without significant impacts on the probability of severe disability in IADLs and self-reported unhealth. In Table 7B, further conditional on the output and health effects, formal caregiving hours have decreased by 4.33% after the introduction of LTCI. The substitution effect of formal care is also validated. However, the positive impacts of LTCI on the two variables of informal caregiving are not statistically significant. There is no evidence to support the substitution effect of informal care. Perhaps because all three effects are relatively small, the overall impacts on hospital expenditures are not significant, as shown in Figure 5E.

## Conclusion and discussion

This study examines the effects of LTCI on hospital expenditures among ineligible disabled individuals from both theoretical and empirical perspectives. Theoretically, based on Becker’s household production function, we find that LTCI will reduce the needed nonmarket time *TF* for the ineligible to produce one unit of *HA*, thus exerting impacts on the inpatient expenditures of ineligible disabled individuals through the substitution, output, and health effects. Empirically, using nationally representative survey data from CHARLS and statistical data from the China City Statistical Yearbook and applying the DID approach, we demonstrate that the implementation of LTCI in the first round of national pilot cities has reduced the number of hospitalizations, the total inpatient expenditure, and the OOP inpatient expenditure among ineligible disabled individuals. However, the impact of LTCI on hospital admission remains ambiguous.

Our findings of the indirect effects of LTCI on hospital expenditures among ineligible disabled individuals add new theoretical explanations and empirical evidence for the indirect impacts of government public policies on ineligible groups. Angelucci and Giorgi (20) and Huang and Zhang (21) have been concerned about this issue and provided empirical evidence, but have not conducted a theoretical analysis. Angelucci and Giorgi (20) find in their study of a poverty alleviation program in Mexico that cash subsidies to impoverished households have not only significantly increased their consumption but also improved the consumption of ineligible households in the same village, by increasing their received loans and transfers from relatives and friends, and by reducing their savings. Huang and Zhang (21) find that the implementation of the New Rural Pension Scheme in China has not only affected the household income, food expenses, labor supply, and overall health status of the age-eligible group (rural older adults aged 60 and above) but also significantly reduces the probability of doing farmwork among the age-ineligible group (rural residents aged 45–60), and also increases their likelihood of engaging in non-farmwork, shifting them from the agricultural to the non-agricultural sector.

We find that the effects of LTCI on the hospital expenditures of ineligible disabled individuals appear to be greater in groups who are aged below 60 or live in urban areas. The explanation may be that, compared to those aged 60 and above, the middle-aged group tends to be healthier on average. Additionally, their family members, especially spouses and children, who are the primary caregivers, are generally younger and face relatively higher time constraints. Given that the real prices of LTC services remain unchanged and the increased efficiency of non-market time, this group experiences a more significant reduction in the shadow price of LTC. As a result, they are more likely to reduce their hospital expenses by increasing the use of formal care services and improving their overall health level. Furthermore, LTCI in most pilot cities (7 in 9) in our sample only covers UEBMI enrollees. Resources were definitely invested more in urban areas at this initial stage, enabling urban residents to benefit more from the increased efficiency of non-market time.

We also find that the impacts of LTCI on the hospital expenditures of ineligible disabled individuals are observed primarily in the policy designs which cover only UEBMI, benefit a larger disabled population, draw up higher reimbursement ceilings, or provide benefits only in kind. These results are in line with those of Lei et al. (22), who report that the beneficial effects of LTCI on the well-being of older adults and their families are primarily observed in the schemes that provided benefits to individuals with moderate disability or dementia, as well as those with severe disability, or had higher reimbursement ceilings. In addition, previous studies have reported heterogeneous effects of LTCI on labor supply and informal care across schemes providing benefits in kind or cash. Fu et al. (23) find that the introduction of LTCI in Japan in 2000 significantly increased labor participation of caregivers, which is opposite to the results of Geyer and Korfhage (24), who report that the implementation of LTCI in Germany in 1995 had a significant negative impact on male labor participation. The reason lies in the fact that LTCI in Germany provides benefits both in kind and cash, while in Japan, on the other hand, it offers only benefits in kind. Courbage et al. (25) find that public LTC support decreases informal caregiving in Spain while increasing it in Italy, which is attributed to different typologies of public LTC coverage. In Spain, benefits are paid conditional on formal care consumption, while benefits in cash are provided without restrictions in Italy.

Concerning mechanisms, we verify that LTCI influences the hospital expenditures of ineligible disabled individuals through the output, health, and substitution effect of formal care, but find no evidence to support the substitution effect of informal care. Several previous studies have discussed the mechanisms. Costa-Font et al. (3) find that the LTC system reform has affected hospital utilization through four channels, including an increased use of outpatient services, improvement in mental health, reduction in loneliness, and housing adjustments. Wang and Feng (5) find through a literature review that the effects of LTCI on medical expenses can be classified into two groups: the negative group includes substitution and health effects, while the positive group contains income and knowledge effects. Moreover, they empirically verify that LTCI has improved the health status of older adults with lower levels of disability and transferred them from hospital care to home care, resulting in a health effect and a substitution effect. Our mechanism analysis of the substitution, output, and health effects in this study provides a meaningful supplement to existing research, theoretically and empirically.

This study has several limitations. First, because the latest wave of CHARLS in 2020 did not provide information on hospital expenditures, we only have one wave of data following the pilot of LTCI. As a result, we can only demonstrate the short-term indirect effects of LTCI on the hospital expenditures of ineligible disabled individuals. Consequently, the long-term impacts remain unknown, which may be a concern in future studies. Second, we have eliminated all respondents with difficulties in ADLs and enrolled in LTCI-covered public medical insurance schemes, because we cannot determine their eligibility for benefits based on the existing information. However, note that this eligible group has only 73 observations in total, and the missed ineligible ones will be even fewer. Thus, the impact of their omission is likely minimal. Moreover, we find that the effects of LTCI on hospital expenditures among ineligible disabled individuals seem to be much larger in the subsample with difficulties in ADLs than in IADLs. This omission, therefore, is likely to lead to an underestimation of the impacts rather than an overestimation.

Overall, this study demonstrates that the introduction of LTCI in the first round of national pilot cities has reduced hospital expenditures among ineligible disabled individuals through the output, health effects, and the substitution effect of formal care. These findings provide both theoretical and empirical evidence for the stepwise expansion and nationwide coverage of LTCI in China. They may also have policy implications for establishing and developing LTC systems in other middle-income and developing countries confronted with increasing demand for LTC. Additionally, this study provides evidence for the indirect impacts of government public policies on groups not directly eligible for those benefits. Consequently, when designing, implementing, and evaluating these policies, it is essential to include the indirect effects on ineligible groups by examining the intent-to-treat effects, rather than solely focusing on the direct impacts on the treatment group.

## Data Availability

The original contributions presented in the study are included in the article/Supplementary material, further inquiries can be directed to the corresponding author.
