# Supplementary material for: Indirect effects of long-term care insurance: does it affect the hospital expenditures of ineligible disabled individuals
Source: Front Public Health. 2025 Nov 5;13:1687682. doi: 10.3389/fpubh.2025.1687682 (PMC12627015; doi:10.3389/fpubh.2025.1687682)
Supplement: Supplementary file 1 [file Data_Sheet_1.PDF]

# Supplementary tables

**Table S1 Policy designs in the first round of national pilot cities**

| City | Covered public medical insurance schemes | Eligibility criteria | Dependency assessment scale |
|---|---|---|---|
| Chengde | UEBMI | Severe | Barthel Index (BI) |
| Changchun | UEBMI +URBMI | Severe and moderate | BI |
| Qiqihaer | UEBMI | Severe | BI |
| Shanghai | UEBMI +URRBMI | Severe, moderate, and mild | Shanghai Unified Needs Assessment Standards for Elderly Care (Trial) |
| Suzhou | UEBMI +URRBMI | Severe and moderate | Suzhou Disability Level Assessment Parameter Table (Trial) |
| Nantong | UEBMI +URBMI | Severe and moderate | BI |
| Ningbo | UEBMI | Severe | BI |
| Anqing | UEBMI | Severe | BI |
| Shangrao | UEBMI | Severe | Shangrao Unified Needs Assessment Standards for Long-term Care |
| Jingmen | UEBMI +URRBMI | Severe | BI |
| Guangzhou | UEBMI | Severe | BI |
| Chongqing | UEBMI | Severe | BI |
| Chengdu | UEBMI | Severe | Chengdu Long-term Care Insurance Comprehensive Assessment Specification for Adult Disability |
| Shihezi | UEBMI +URRBMI | Severe | BI |
| Qingdao | UEBMI +URRBMI | Severe and moderate | BI |

**Table S2 Robustness: applying the staggered DID**

| Variables | (1)<br>Hospital admission | (2)<br>Number of hospitalizations | (3)<br>Ln (Total inpatient expenditure) | (4)<br>Ln (OOP inpatient expenditure) |
|---|---|---|---|---|
| Treat×Post | -0.0480*** | -0.0619 | -0.531*** | -0.419*** |
| | (0.0174) | (0.0378) | (0.163) | (0.139) |
| Observations | 15160 | 15160 | 14758 | 14758 |
| R-squared | 0.011 | 0.012 | 0.014 | 0.012 |

Notes: Standard errors clustered at the city level are reported in parentheses. ***, **, and * mean the significance levels of 1%, 5%, and 10% respectively. Each regression controls for individual covariates, city-level covariates, individual FE, and year FE. Individual and city-level covariates are the same as in Table 2.

**Table S3 Robustness: Placebo tests by changing pilot year**

| Variables | (1) Hospital admission | (2) Number of hospitalizations | (3) Ln (Total inpatient expenditure) | (4) Ln (OOP inpatient expenditure) |
|---|---|---|---|---|
| Panel A: Assuming that the pilot was implemented in 2015 | | | | |
| $Treat_{ic}\times Post_t$ | 0.0402 | 0.0243 | 0.224 | 0.190 |
| | (0.0379) | (0.0662) | (0.307) | (0.248) |
| Observations | 13156 | 13156 | 12789 | 12789 |
| R-squared | 0.012 | 0.013 | 0.015 | 0.014 |
| Panel B: Assuming that the pilot was implemented in 2013 | | | | |
| $Treat_{ic}\times Post_t$ | 0.0121 | -0.0138 | 0.295 | 0.225 |
| | (0.0527) | (0.0661) | (0.409) | (0.362) |
| Observations | 13156 | 13156 | 12789 | 12789 |
| R-squared | 0.012 | 0.013 | 0.015 | 0.014 |

Notes: Standard errors clustered at the city level are reported in parentheses. ***, **, and * mean the significance levels of 1%, 5%, and 10% respectively. Each regression controls for individual covariates, city-level covariates, individual FE, and year FE. Individual and city-level covariates are the same as in Table 2.

## Supplementary figures

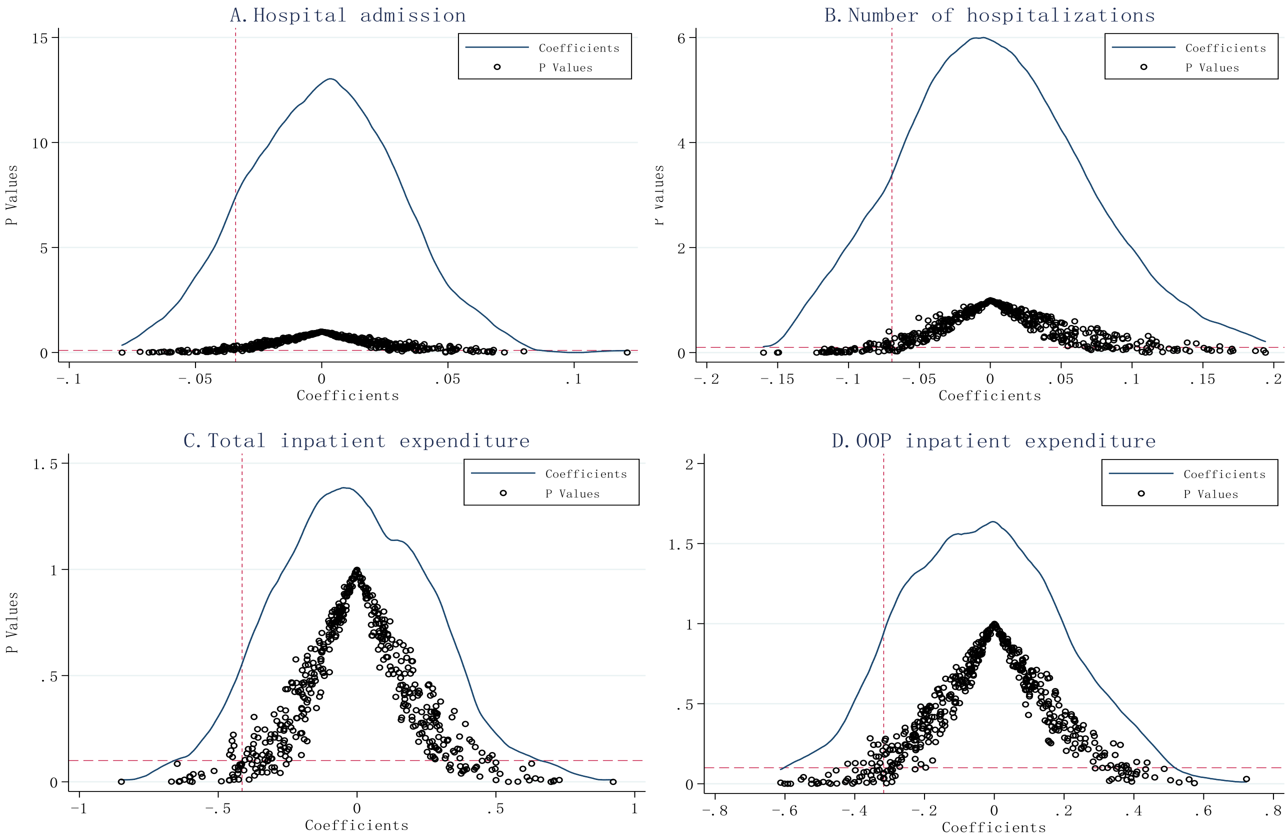


**Figure S1 Robustness: Placebo tests by randomly generating the treatment group**

Notes: The X-axis represents the estimated coefficients, while the Y-axis indicates the corresponding P values of these coefficients. Each regression controls for individual covariates, city-level covariates, individual FE, and year FE. Individual and city-level covariates are the same as in Table 2.
